# Author Correction: Limited generalizability of multivariate brain-based dimensions of child psychiatric symptoms

**DOI:** 10.1038/s44271-024-00078-5

**Published:** 2024-04-06

**Authors:** Bing Xu, Lorenza Dall’Aglio, John Flournoy, Gerda Bortsova, Brenden Tervo-Clemmens, Paul Collins, Marleen de Bruijne, Monica Luciana, Andre Marquand, Hao Wang, Henning Tiemeier, Ryan L. Muetzel

**Affiliations:** 1 Department of Child and Adolescent Psychology and Psychiatry, Erasmus MC University Medical Center Rotterdam-Sophia Children’s Hospital, Rotterdam, The Netherlands; 2 The Generation R Study Group, Erasmus MC University Medical Center Rotterdam, Rotterdam, The Netherlands; 3 Department of Psychology, Harvard University, Cambridge, MA USA; 4 Department of Radiology and Nuclear Medicine, Biomedical Imaging Group Rotterdam, Erasmus MC University Medical Center Rotterdam, Rotterdam, The Netherlands; 5 Department of Psychiatry, Massachusetts General Hospital, Harvard Medical School, Boston, MA USA; 6 Department of Psychology, University of Minnesota, Minneapolis, MN USA; 7 Department of Computer Science, University of Copenhagen, Copenhagen, Denmark; 8 Donders Institute for Brain, Cognition and Behaviour, Radboud University, Nijmegen, The Netherlands; 9 Radboud University Medical Center, Nijmegen, The Netherlands; 10 Leiden Institute of Advanced Computer Science, Leiden University, Leiden, The Netherlands; 11 Department of Social and Behavioral Sciences, Harvard T. Chan School of Public Health, Boston, MA USA; 12 Department of Radiology and Nuclear Medicine, Erasmus MC University Medical Center Rotterdam, Rotterdam, The Netherlands

**Keywords:** Predictive markers, Magnetic resonance imaging

Correction to: *Communications Psychology* 10.1038/s44271-024-00063-y, published online 28 February 2024

In this article the graphs in Figure 3 showing the top 20% of the connectivity patterns, i.e., those in panels Figure 3a (CV 1, attention problems) and Figure 3b (CV2, aggression, rule-breaking) were inadvertently swapped. The original article has been corrected.

