# Supplementary material for: Limited generalizability of multivariate brain-based dimensions of child psychiatric symptoms
Source: Commun Psychol. 2024 Feb 28;2:16. doi: 10.1038/s44271-024-00063-y (PMC11332032; doi:10.1038/s44271-024-00063-y)
Supplement: Supplementary file 1 — Supplementary information [file 44271_2024_63_MOESM1_ESM.pdf]

Supplementary Information

Limited generalizability of multivariate brain-based dimensions of child psychiatric symptoms

Supplementary Methods

Supplementary Tables 1-11

Supplementary Figures 1-3

Supplementary References

Supplementary Methods

Clustering

To further explore the utility of the brain-based dimension we found, we also examined the possibility of clustering, often known as biotypes, using the brain-based dimensions we found. In the current study, the selected brain canonical variates were further used to explore potential classifications of participants with similar patterns of resting-state functional connectivity. Hierarchical clustering with Euclidean distance measure and Ward's D linkage method was first used, and the optimal number of clusters was selected based on CH index (variance ratio criterion) and silhouette index[1]. Besides the visual inspection of these two indices, we also performed a permutation-based test to check the statistical significance of these two indices based on a previous study[2].

Supplementary Table 1
MRI acquisition parameters in ABCD

	Matrix	Slices	FOV	TR (ms)	TE (ms)	TI (ms)	Flip Angle (deg)	MultiBand Acceleration	Acquisition Time
**Siemens**									
**T1**	256 × 256	176	256 × 256	2500	2.88	1060	8	Off	7:12
**fMRI**	90 × 90	60	216 × 216	800	30	N/A	52	6	
**Philips**									
**T1**	256 × 256	225	256 × 240	6.31	2.9	1060	8	Off	5:38
**fMRI**	90 × 90	60	216 × 216	800	30	N/A	52	6	
**GE**									
**T1**	256 × 256	208	256 × 256	2500	2	1060	8	Off	6:09
**fMRI**	90 × 90	60	216 × 216	800	30	N/A	52	6	

Note. Parameters retrieved from the ABCC collection (https://collection3165.readthedocs.io/en/stable/inputs/).

Supplementary Table 2
MRI acquisition parameters in Generation R

	Matrix	Slices thickness (mm)/ Number of slices	FOV	TR (ms)	TE (ms)	TI (ms)	Flip Angle (deg)
**GE 750**							
**T1**	220 × 220	1.0/230	220 × 220	8.77	3.4	600	10
**fMRI**	64 × 64	4.0/36	216 × 216	1760	30	N/A	85

Note. T1-weighted images were obtained using a coronal inversion recovery fast spoiled gradient recalled sequence using ARC acceleration (GE option BRAVO). Rs-fMRI data were obtained using an interleaved axial echo planar imaging sequence. Total duration of the resting-state scan was 5 minutes 52 seconds for each child.

Supplementary Table 3

Canonical correlations in ABCD and Generation R across 30 splits

	Canonical Correlations	ABCD (n = 4,892) Training	Test	p_{fdr}	Sparsity		Generation R n = 2,043
		n=4,255	*n=637*				
Split 1	r_1	0.23	0.13**	**0.005**	rs-fMRI	0.6	0.02
	r_2	0.23	0.01	0.81	CBCL	0.5	0.03
	r_3	0.19	0.08	0.13			0.02
		n=4,092	*n=800*				
Split 2	r_1	0.23	0.13***	**<0.001**	rs-fMRI	0.7	0.02
	r_2	0.23	0.09*	**0.035**	CBCL	0.5	0.01
	r_3	0.21	0.10*	**0.012**			0.002
		n=4,295	*n=597*				
Split 3	r_1	0.23	0.08	0.20	rs-fMRI	0.6	0.03
	r_2	0.22	0.07	0.20	CBCL	0.5	0.04
	r_3	0.21	0.01	0.82			0.01
		n=3,976	*n=916*				
Split 4	r_1	0.24	0.15***	**<0.001**	rs-fMRI	0.9	0.01
	r_2	0.24	0.09*	**0.014**	CBCL	0.5	0.02
	r_3	0.22	0.06	0.10			0.01
		n=4,167	*n=725*				
Split 5	r_1	0.24	0.11*	**0.015**	rs-fMRI	0.8	0.03
	r_2	0.24	0.02	0.82	CBCL	0.5	0.04
	r_3	0.21	0.07	0.18			0.001
		n=4,401	*n=491*				
Split 6	r_1	0.20	0.07	0.30	rs-fMRI	0.4	0.02
	r_2	0.19	0.07	0.30	CBCL	0.5	0.001
	r_3	0.18	0.06	0.30			0.03
		n=4,229	*n=663*				
Split 7	r_1	0.23	0.14**	**0.003**	rs-fMRI	0.6	0.01
	r_2	0.22	0.12***	**<0.001**	CBCL	0.5	0.02
	r_3	0.20	0.05	0.19			0.02
		n=4,139	*n=753*				
Split 8	r_1	0.22	0.16***	**<0.001**	rs-fMRI	0.6	0.03
	r_2	0.21	0.10*	**0.018**	CBCL	0.5	0.02
	r_3	0.21	0.04	0.29			0.02
		n=3,811	*n=1,081*				
Split 9	r_1	0.23	0.19***	**<0.001**	rs-fMRI	0.8	0.01
	r_2	0.24	0.16***	**<0.001**	CBCL	0.5	0.01
	r_3	0.21	0.08*	**0.015**			0.01
		n=4,119	*n=773*				
Split 10	r_1	0.24	0.10*	**0.025**	rs-fMRI	0.5	0.01
	r_2	0.23	0.08	0.05	CBCL	0.5	0.02
	r_3	0.19	0.04	0.28			0.01

		n=4,194	*n=698*				
Split 11	r_1	0.22	0.10*	**0.018**	rs-fMRI	0.6	0.003
	r_2	0.22	0.11*	**0.010**	CBCL	0.5	0.02
	r_3	0.19	0.07	0.08			0.01
		n=4,416	*n=476*				
Split 12	r_1	0.23	0.07	0.38	rs-fMRI	0.8	0.07*
	r_2	0.23	0.03	0.83	CBCL	0.5	0.04
	r_3	0.21	0.06	0.38			0.06
		n=4,230	*n=662*				
Split 13	r_1	0.23	0.15**	**0.003**	rs-fMRI	0.7	0.05
	r_2	0.24	0.13***	**<0.001**	CBCL	0.5	0.04
	r_3	0.20	0.09*	**0.041**			0.02
		n=4,206	*n=686*				
Split 14	r_1	0.24	0.12***	**<0.001**	rs-fMRI	0.7	0.01
	r_2	0.22	0.01	0.94	CBCL	0.7	0.03
	r_3	0.21	0.04	0.50			0.01
		n=4,198	*n=694*				
Split 15	r_1	0.23	0.14**	**<0.001**	rs-fMRI	0.7	0.02
	r_2	0.24	0.09*	**0.038**	CBCL	0.5	0.04
	r_3	0.21	0.07	0.08			0.004
		n=4,118	*n=774*				
Split 16	r_1	0.24	0.10*	**0.040**	rs-fMRI	0.8	0.05
	r_2	0.22	0.06	0.15	CBCL	0.5	0.04
	r_3	0.21	0.01	0.76			0.06
		n=4,057	*n=835*				
Split 17	r_1	0.22	0.13**	**<0.001**	rs-fMRI	0.5	0.05
	r_2	0.22	0.07	0.09	CBCL	0.5	0.01
	r_3	0.19	0.02	0.52			0.03
		n=4,055	*n=837*				
Split 18	r_1	0.21	0.12**	**0.003**	rs-fMRI	0.7	0.05
	r_2	0.17	0.06	0.14	CBCL	0.6	0.01
	r_3	0.18	0.002	0.95			0.01
		n=3,784	*n=1,108*				
Split 19	r_1	0.23	0.18***	**<0.001**	rs-fMRI	0.8	0.03
	r_2	0.25	0.13***	**<0.001**	CBCL	0.5	0.05
	r_3	0.22	0.08*	**0.010**			0.01
		n=4,154	*n=738*				
Split 20	r_1	0.24	0.10*	**0.025**	rs-fMRI	0.6	0.04
	r_2	0.22	0.06	0.34	CBCL	0.5	0.06
	r_3	0.22	0.02	0.74			0.05
		n=4,410	*n=482*				
Split 21	r_1	0.24	0.05	0.40	rs-fMRI	0.7	0.02
	r_2	0.25	0.04	0.40	CBCL	0.5	0.03
	r_3	0.21	0.04	0.40			0.02
		n=4,141	*n=751*				
Split 22	r_1	0.23	0.17***	**<0.001**	rs-fMRI	0.8	0.003
	r_2	0.24	0.11**	**0.008**	CBCL	0.5	0.02
	r_3	0.20	0.05	0.28			0.01
		n=4,178	*n=714*				
Split 23	r_1	0.23	0.08	0.14	rs-fMRI	0.7	0.05
	r_2	0.24	0.05	0.34	CBCL	0.5	0.05
	r_3	0.22	0.02	0.70			0.01
		n=4,278	*n=614*				
Split 24	r_1	0.22	0.08	0.20	rs-fMRI	0.5	0.03
	r_2	0.22	0.03	0.47	CBCL	0.5	0.06
	r_3	0.20	0.04	0.47			0.04

		n=4,194	*n=698*				
Split	r_1	0.19	0.13***	**<0.001**	rs-fMRI	0.3	0.03
25	r_2	0.17	0.04	0.33	CBCL	0.5	0.03
	r_3	0.15	0.05	0.28			0.02
		n=4,338	*n=554*				
Split	r_1	0.23	0.15**	**0.005**	rs-fMRI	0.8	0.01
26	r_2	0.25	0.06	0.24	CBCL	0.5	0.05
	r_3	0.20	0.07	0.16			0.03
		n=3,815	*n=1,077*				
Split	r_1	0.23	0.18***	**<0.001**	rs-fMRI	0.8	0.04
27	r_2	0.23	0.12***	**<0.001**	CBCL	0.5	0.04
	r_3	0.21	0.09**	**0.006**			0.03
		n=4,164	*n=728*				
Split	r_1	0.22	0.10**	**<0.001**	rs-fMRI	0.4	0.02
28	r_2	0.12	0.01	0.77	CBCL	0.6	0.01
	r_3	0.12	0.04	0.46			0.05
		n=4,079	*n=813*				
Split	r_1	0.25	0.14***	**<0.001**	rs-fMRI	0.7	0.002
29	r_2	0.21	0.04	0.34	CBCL	0.7	0.02
	r_3	0.18	0.03	0.42			0.02
		n=4,342	*n=550*				
Split	r_1	0.24	0.15**	**0.006**	rs-fMRI	0.9	0.005
30	r_2	0.23	0.14**	**<0.001**	CBCL	0.5	0.001
	r_3	0.18	0.02	0.86			0.05

Note. We first completed the weighted PCA, penalty parameter search, and fitting the SCCA model in the $ABCD_{Training}$ set. Next, the PCA eigenvectors we derived from the $ABCD_{Training}$ sets were applied to brain connectivity data in the $ABCD_{Test}$ sets to derive brain PCs in $ABCD_{Test}$ sets, then the SCCA weight vectors from $ABCD_{Training}$ sets were projected to the brain PCs and CBCL scores in the $ABCD_{Test}$ sets. In this way, the canonical correlations were computed, and the significance of the $ABCD_{Test}$ canonical correlations was assessed by permutation tests. P values were corrected for multiple testing by False Discovery Rate (FDR). All the p values of $ABCD_{Training}$ sets were smaller than 0.001. All the p values of Generation R were larger than 0.05 except for split 12 ($p_{r1genr} = 0.01$). The bold p values were smaller than 0.05. This process was repeated 30 times in the 30 training-test splits.

* $p < 0.05$, ** $p < 0.01$, *** $p < 0.001$

Supplementary Table 4

Canonical correlations in training and test sets of ABCD across 10 splits (Train-test split with pooled multisite data)

	Canonical Correlations	Training	Test	Sparsity	
Split 1	r_1	0.24	0.12***	Rs-fMRI	0.7
	r_2	0.24	0.08*	CBCL	0.5
	r_3	0.22	0.04		
Split 2	r_1	0.22	0.12***	Rs-fMRI	0.6
	r_2	0.22	0.07*	CBCL	0.5
	r_3	0.19	0.03		
Split 3	r_1	0.24	0.15***	Rs-fMRI	0.8
	r_2	0.23	0.13***	CBCL	0.5
	r_3	0.21	0.04		
Split 4	r_1	0.22	0.18***	Rs-fMRI	0.5
	r_2	0.21	0.13**	CBCL	0.5
	r_3	0.19	0.06		
Split 5	r_1	0.23	0.08*	Rs-fMRI	0.6
	r_2	0.24	0.06	CBCL	0.5
	r_3	0.20	0.05		
Split 6	r_1	0.25	0.14***	Rs-fMRI	0.8
	r_2	0.23	0.07*	CBCL	0.5
	r_3	0.26	0.09**		
Split 7	r_1	0.25	0.12***	Rs-fMRI	0.7
	r_2	0.23	0.04	CBCL	0.5
	r_3	0.24	0.06		
Split 8	r_1	0.25	0.15***	Rs-fMRI	0.8
	r_2	0.21	0.10***	CBCL	0.5
	r_3	0.23	0.13**		
Split 9	r_1	0.23	0.13***	Rs-fMRI	0.6
	r_2	0.21	0.08**	CBCL	0.5
	r_3	0.24	0.10**		
Split 10	r_1	0.24	0.14***	Rs-fMRI	0.8
	r_2	0.25	0.09**	CBCL	0.5
	r_3	0.21	0.10**		

Note. We pooled the data from 21 sites together and randomly split it into a training set ($ABCD_{Training}$, 80% of the data) and a test set ($ABCD_{Test}$, 20% of the data). The canonical correlations in the $ABCD_{Test}$ sets were calculated by applying the weight vectors obtained from the $ABCD_{Training}$ set SCCA model to the test sets. This process was repeated 10 times in the 10 training-test splits.

* $p < 0.05$, ** $p < 0.01$, *** $p < 0.001$

Supplementary Table 5

Standard PCA followed by sparse CCA (5-fold cross validation without duplicated study sites)

	Canonical Correlations	Training set	Test set	Generation R
Split 1	r_1	0.22	0.10**	0.03
	r_2	0.18	0.06	0.02
	r_3	0.17	0.02	0.04
Split 2	r_1	0.21	0.06	0.05
	r_2	0.18	0.01	0.03
	r_3	0.17	0.02	0.003
Split 3	r_1	0.24	0.11***	0.01
	r_2	0.20	0.08*	0.02
	r_3	0.19	0.08*	0.02
Split 4	r_1	0.22	0.11***	0.002
	r_2	0.20	0.11***	0.001
	r_3	0.17	0.06	0.02
Split 5	r_1	0.23	0.11**	0.02
	r_2	0.18	0.10**	0.04
	r_3	0.21	0.04	0.04

Note. We performed the standard PCA instead of weighted PCA in the $ABCD_{Training}$ sets and GenR, and adopted 5-fold cross validation, in which no duplicated study sites were assigned to $ABCD_{Test}$ sets.
* $p < 0.05$, ** $p < 0.01$, *** $p < 0.001$

Supplementary Table 6

Standard PCA followed by traditional CCA (5-fold cross validation without duplicated study sites)

	Canonical Correlations	Training set	Test set	Generation R
Split 1	r_1	0.26	0.05	0.002
	r_2	0.21	0.07	0.05
	r_3	0.20	0.03	0.03
Split 2	r_1	0.26	0.10^{**}	0.02
	r_2	0.23	0.04	0.01
	r_3	0.19	0.02	0.01
Split 3	r_1	0.25	0.06	0.01
	r_2	0.22	0.02	0.002
	r_3	0.18	0.01	0.002
Split 4	r_1	0.25	0.07^{*}	0.01
	r_2	0.23	0.05	0.01
	r_3	0.19	0.01	0.01
Split 5	r_1	0.25	0.10^{**}	0.02
	r_2	0.22	0.08^{*}	0.03
	r_3	0.18	0.05	0.05

Note. We performed the standard PCA instead of weighted PCA and traditional CCA without sparsity in the $ABCD_{Training}$ sets and GenR, using 5-fold cross validation in ABCD.
* $p < 0.05$, ** $p < 0.01$, *** $p < 0.001$

Supplementary Table 7

Canonical correlations in ABCD and Generation R across 10 splits (50 brain PCs)

	ABCD (n = 4,892)					Generation R n = 2,043
	**Canonical Correlations**	**Training**	**Test**	**Sparsity**		
		n=4,255	*n=637*			
Split 1	r_1	0.20	0.15***	rs-fMRI	0.8	0.05
	r_2	0.18	0.08	CBCL	0.5	0.04
	r_3	0.16	0.04			0.09*
		n=4,092	*n=800*			
Split 2	r_1	0.20	0.13***	rs-fMRI	0.7	0.01
	r_2	0.19	0.10**	CBCL	0.5	0.001
	r_3	0.15	0.08			0.005
		n=4,295	*n=597*			
Split 3	r_1	0.21	0.06	rs-fMRI	0.8	0.07*
	r_2	0.19	0.03	CBCL	0.5	0.04
	r_3	0.17	0.03			0.05
		n=3,976	*n=916*			
Split 4	r_1	0.19	0.15***	rs-fMRI	0.9	0.02
	r_2	0.20	0.11***	CBCL	0.5	0.001
	r_3	0.16	0.07			0.003
		n=4,167	*n=725*			
Split 5	r_1	0.21	0.13**	rs-fMRI	0.9	0.003
	r_2	0.20	0.06	CBCL	0.5	0.007
	r_3	0.16	0.08			0.002
		n=4,401	*n=491*			
Split 6	r_1	0.20	0.09*	rs-fMRI	0.7	0.06*
	r_2	0.19	0.09	CBCL	0.5	0.07*
	r_3	0.17	0.08			0.08*
		n=4,229	*n=663*			
Split 7	r_1	0.20	0.14***	rs-fMRI	0.7	0.01
	r_2	0.19	0.15**	CBCL	0.5	0.02
	r_3	0.17	0.07			0.02
		n=4,139	*n=753*			
Split 8	r_1	0.20	0.17***	rs-fMRI	0.8	0.06*
	r_2	0.19	0.10**	CBCL	0.5	0.06
	r_3	0.16	0.05			0.03
		n=3,811	*n=1,081*			
Split 9	r_1	0.19	0.20***	rs-fMRI	0.9	0.01
	r_2	0.18	0.14***	CBCL	0.5	0.01
	r_3	0.18	0.08**			0.04
		n=4,119	*n=773*			
Split 10	r_1	0.20	0.15***	rs-fMRI	0.7	0.11***
	r_2	0.18	0.11**	CBCL	0.5	0.06
	r_3	0.17	0.09*			0.12***

Note. The significance of canonical correlations was assessed by permutation tests.
P values were corrected for multiple testing by False Discovery Rate (FDR).
* $p < 0.05$, ** $p < 0.01$, *** $p < 0.001$

Supplementary Table 8

Canonical correlations in ABCD and Generation R across 10 splits (200 brain PCs)

	ABCD (n = 4,892)					Generation R n = 2,043
	Canonical Correlations	Training	Test	Sparsity		
		n=4,255	*n=637*			
Split 1	r_1	0.29	0.12***	rs-fMRI	0.8	0.03
	r_2	0.27	0.05	CBCL	0.5	0.04
	r_3	0.30	0.02			0.06
		n=4,092	*n=800*			
Split 2	r_1	0.31	0.15***	rs-fMRI	0.8	0.02
	r_2	0.28	0.08*	CBCL	0.5	0.06*
	r_3	0.29	0.14***			0.004
		n=4,295	*n=597*			
Split 3	r_1	0.29	0.08*	rs-fMRI	0.7	0.05
	r_2	0.26	0.08	CBCL	0.5	0.05
	r_3	0.29	0.04			0.03
		n=3,976	*n=916*			
Split 4	r_1	0.29	0.16***	rs-fMRI	0.7	0.01
	r_2	0.28	0.12**	CBCL	0.5	0.01
	r_3	0.28	0.06			0.01
		n=4,167	*n=725*			
Split 5	r_1	0.29	0.14***	rs-fMRI	0.8	0.003
	r_2	0.30	0.07	CBCL	0.5	0.01
	r_3	0.26	0.06			0.02
		n=4,401	*n=491*			
Split 6	r_1	0.24	0.06	rs-fMRI	0.4	0.04
	r_2	0.24	0.07	CBCL	0.5	0.07*
	r_3	0.22	0.08			0.06
		n=4,229	*n=663*			
Split 7	r_1	0.29	0.13**	rs-fMRI	0.7	0.06*
	r_2	0.26	0.11**	CBCL	0.5	0.06*
	r_3	0.30	0.06			0.04
		n=4,139	*n=753*			
Split 8	r_1	0.26	0.19***	rs-fMRI	0.5	0.04
	r_2	0.24	0.09**	CBCL	0.5	0.04
	r_3	0.26	0.03			0.06*
		n=3,811	*n=1,081*			
Split 9	r_1	0.29	0.18***	rs-fMRI	0.7	0.02
	r_2	0.29	0.11**	CBCL	0.5	0.01
	r_3	0.28	0.08*			0.001
		n=4,119	*n=773*			
Split 10	r_1	0.30	0.11*	rs-fMRI	0.6	0.06*
	r_2	0.26	0.11*	CBCL	0.5	0.06*
	r_3	0.27	0.06			0.03

Note. The significance canonical correlations was assessed by permutation tests.
P values were corrected for multiple testing by False Discovery Rate (FDR).
* p < 0.05, ** p < 0.01, *** p < 0.001

Supplementary Table 9

Descriptive statistics of CBCL scores across two cohorts

CBCL syndrome scales	ABCD (n = 4,892)			Generation R (n = 2,043)		
	M(SD) *(raw scores)*	Range *(raw scores)*	M(SD) *(T scores)*	M(SD) *(raw scores)*	Range *(raw scores)*	M(SD) *(T scores)*
Anxious/depressed	2.6(3.1)	(0, 26)	53.5(6.1)	2.2(2.6)	(0, 19)	52.7(5.0)
Withdrawn/depressed	1.0(1.7)	(0, 14)	53.3(5.6)	1.1(1.6)	(0, 10)	53.7(5.6)
Somatic	1.5(1.9)	(0, 15)	54.9(6.0)	1.5(2.0)	(0, 15)	54.8(6.0)
Social	1.5(2.2)	(0, 18)	52.5(4.4)	1.5(2.1)	(0, 16)	52.5(4.3)
Aggressive	3.1(4.2)	(0, 36)	52.6(5.3)	2.7(3.5)	(0, 28)	52.0(4.3)
Rule-breaking	1.1(1.7)	(0, 18)	52.5(4.5)	0.9(1.4)	(0, 11)	52.0(3.6)
Thought problems	1.5(2.1)	(0, 18)	53.6(5.7)	1.5(2.0)	(0, 15)	53.4(5.5)
Attention problems	2.7(3.3)	(0, 20)	53.5(5.8)	3.0(3.0)	(0, 18)	53.6(5.0)
Internalizing problems	5.1(5.6)	(0, 51)	48.4(10.6)	4.7(5.0)	(0, 37)	48.1(9.9)
Externalizing problems	4.1(5.6)	(0, 48)	45.2(10.0)	3.6(4.6)	(0, 38)	44.4(9.2)

Supplementary Table 10

Canonical correlations in ABCD and Generation R across 10 splits (non-harmonized data)

	ABCD					Generation R n = 2,076
	**Canonical Correlations**	**Training**	**Test**	**Sparsity**		
		n=5156	*n=1373*			
Split 1	r_1	0.24	0.11***	rs-fMRI	0.8	0.03
	r_2	0.22	0.09**	CBCL	0.5	0.06
	r_3	0.22	0.06*			0.06
		n=4898	*n=1631*			
Split 2	r_1	0.21	0.17***	rs-fMRI	0.5	0.07**
	r_2	0.22	0.12***	CBCL	0.5	0.002
	r_3	0.19	0.05			0.06*
		n=4613	*n=1916*			
Split 3	r_1	0.18	0.13***	rs-fMRI	0.3	0.02
	r_2	0.15	0.11***	CBCL	0.5	0.03
	r_3	0.17	0.04			0.01
		n=5058	*n=1471*			
Split 4	r_1	0.21	0.12***	rs-fMRI	0.5	0.08***
	r_2	0.21	0.07**	CBCL	0.5	0.04
	r_3	0.21	0.04			0.01
		n=5408	*n=1121*			
Split 5	r_1	0.23	0.09**	rs-fMRI	0.6	0.03
	r_2	0.23	0.06*	CBCL	0.5	0.01
	r_3	0.18	0.07*			0.01
		n=4846	*n=1683*			
Split 6	r_1	0.18	0.14***	rs-fMRI	0.3	0.01
	r_2	0.15	0.09***	CBCL	0.5	0.01
	r_3	0.14	0.06*			0.01
		n=5279	*n=1250*			
Split 7	r_1	0.22	0.15***	rs-fMRI	0.5	0.04
	r_2	0.20	0.08**	CBCL	0.5	0.04
	r_3	0.19	0.06*			0.01
		n=5660	*n=869*			
Split 8	r_1	0.21	0.16***	rs-fMRI	0.5	0.08***
	r_2	0.22	0.02	CBCL	0.5	0.04
	r_3	0.18	0.12***			0.05
		n=4703	*n=1826*			
Split 9	r_1	0.14	0.12***	rs-fMRI	0.2	0.02
	r_2	0.12	0.09***	CBCL	0.7	0.01
	r_3	0.09	0.02			0.01
		n=5209	*n=1320*			
Split 10	r_1	0.20	0.11***	rs-fMRI	0.3	0.01
	r_2	0.17	0.09***	CBCL	0.5	0.03
	r_3	0.16	0.04			0.01

Note. The significance of canonical correlations was assessed by permutation tests.
P values were corrected for multiple testing by False Discovery Rate (FDR).
* $p < 0.05$, ** $p < 0.01$, *** $p < 0.001$

Supplementary Table 11

CBCL scores across two clusters in ABCD

	**ABCD** *N = 4,892*	
	Cluster 1	***Cluster 2***
N	1,181	3,711
Child Behavior Checklist (CBCL), M (SD)		
Anxious/depressed	2.6 (3.2)	2.5 (3.1)
Withdrawn/depressed	1.1 (1.9)	1.0 (1.6)
Somatic	1.6 (2.1)	1.5 (1.9)
Social	1.0 (2.4)	1.4 (2.1)
Aggressive	3.3 (4.6)	3.0 (4.0)
Rule-breaking	1.2 (1.9)	1.0 (1.7)
Thought problems	1.6 (2.3)	1.5 (2.1)
Attention problems	2.9 (3.5)	2.6 (3.3)
Internalizing problems	5.3 (6.0)	5.0 (5.4)
Externalizing problems	4.5 (6.1)	4.0 (5.4)
Total scores	18.4 (19.3)	16.8 (16.7)

Supplementary Figure 1

Canonical correlations and CBCL loadings across 10 splits (different PCs) in ABCD

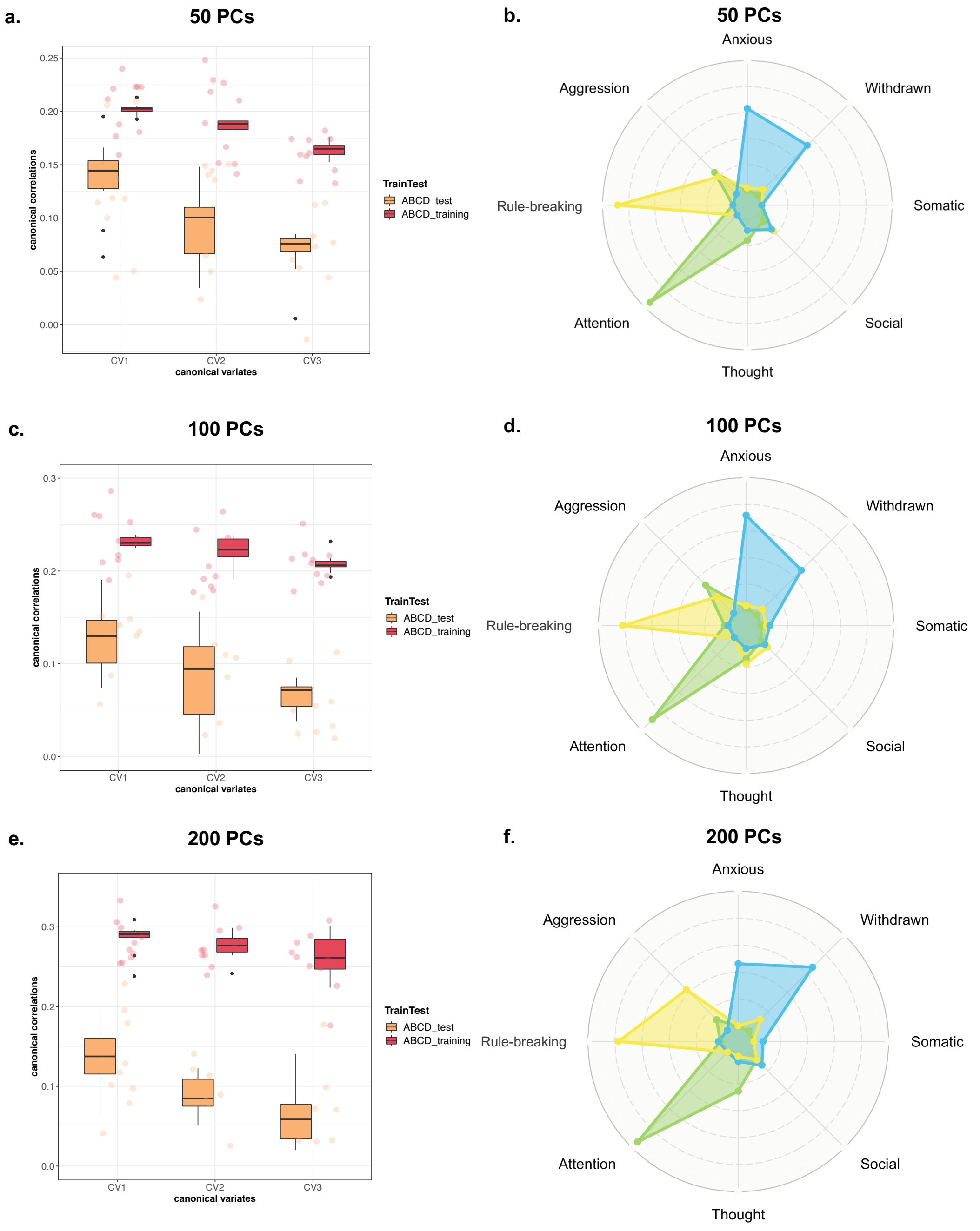

a-b. Canonical correlations and CBCL loadings when we put 50 brain PCs in the SCCA model. In the boxplot, the center line is the median and box limits are the 25th percentile and 75th percentile. **c-d.** Canonical correlations and CBCL loadings when we put 100 brain PCs in the SCCA model. **e-f.** Canonical correlations and CBCL loadings when we put 200 brain PCs in the SCCA model.

Supplementary Figure 2

Canonical loadings in Generation R in qualitative replication

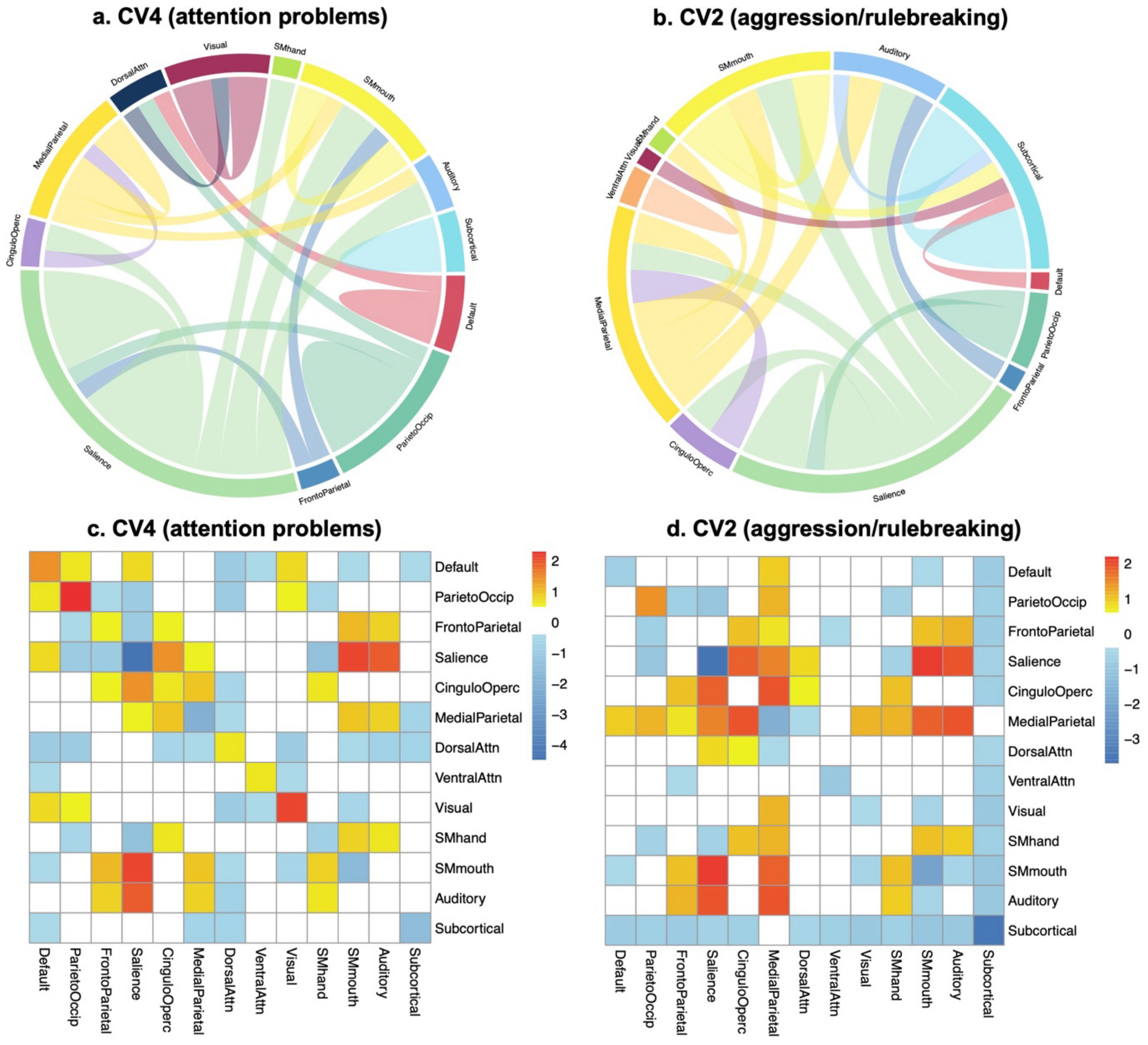

a-b. The top 20% of the connectivity patterns that contributed most for the two canonical variates in Generation R that are similar with ABCD. **c-d.** brain connectivity modules involved in the two identified canonical variates in Generation R, which are not entirely the same with ABCD.

Supplementary Figure 3

Clustering based on brain canonical variate scores in ABCD

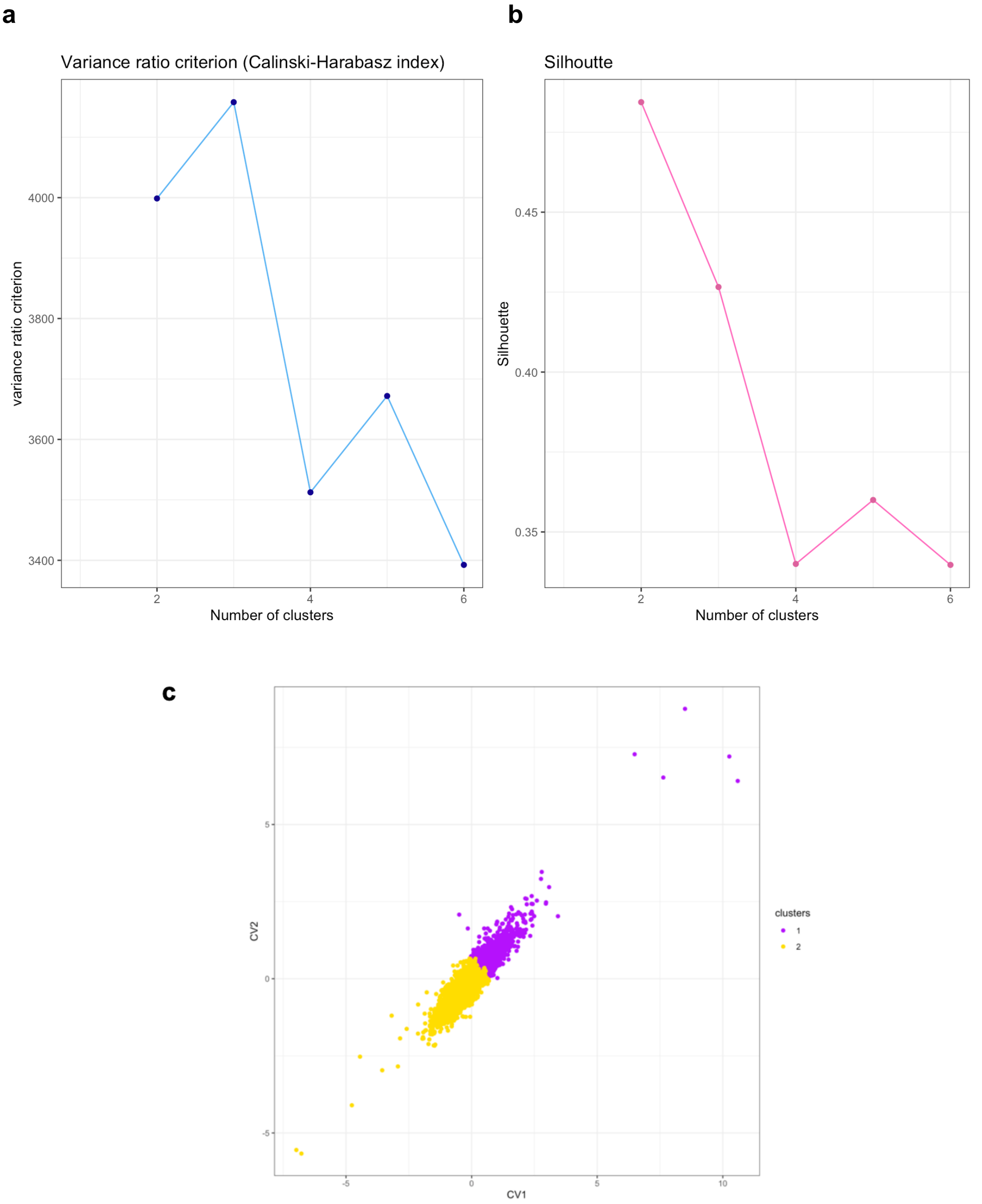

a-b. The variance ratio criterion and the silhouette index showed a two-cluster solution. Both metrics were not statistically significant under null hypothesis of no clusters. **c.** The visualization of the distribution of the two clusters along the two brain canonical variate dimensions.

Supplementary References

1. Dinga, R. *et al.* Evaluating the evidence for biotypes of depression: Methodological replication and extension of. *NeuroImage Clin.* **22**, 101796 (2019).
2. Liu, Y., Hayes, D. N., Nobel, A. & Marron, J. S. Statistical Significance of Clustering for High-Dimension, Low-Sample Size Data. *J. Am. Stat. Assoc.* **103**, 1281–1293 (2008).
