# Supplementary material for: Limited generalizability of multivariate brain-based dimensions of child psychiatric symptoms
Source: Commun Psychol. 2024 Feb 28;2:16. doi: 10.1038/s44271-024-00063-y (PMC11332032; doi:10.1038/s44271-024-00063-y)
Supplement: Supplementary file 3 — Reporting Summary [file 44271_2024_63_MOESM3_ESM.pdf]

nature portfolio

Corresponding author(s): Henning Tiemeier

Last updated by author(s): Jan 15, 2024

Reporting Summary

Nature Portfolio wishes to improve the reproducibility of the work that we publish. This form provides structure for consistency and transparency in reporting. For further information on Nature Portfolio policies, see our Editorial Policies and the Editorial Policy Checklist.

Statistics

For all statistical analyses, confirm that the following items are present in the figure legend, table legend, main text, or Methods section.

n/a	Confirmed	
☐	☒	The exact sample size (*n*) for each experimental group/condition, given as a discrete number and unit of measurement
☐	☒	A statement on whether measurements were taken from distinct samples or whether the same sample was measured repeatedly
☐	☒	The statistical test(s) used AND whether they are one- or two-sided<br>*Only common tests should be described solely by name; describe more complex techniques in the Methods section.*
☐	☒	A description of all covariates tested
☐	☒	A description of any assumptions or corrections, such as tests of normality and adjustment for multiple comparisons
☐	☒	A full description of the statistical parameters including central tendency (e.g. means) or other basic estimates (e.g. regression coefficient) AND variation (e.g. standard deviation) or associated estimates of uncertainty (e.g. confidence intervals)
☐	☒	For null hypothesis testing, the test statistic (e.g. *F*, *t*, *r*) with confidence intervals, effect sizes, degrees of freedom and *P* value noted<br>*Give P values as exact values whenever suitable.*
☒	☐	For Bayesian analysis, information on the choice of priors and Markov chain Monte Carlo settings
☐	☒	For hierarchical and complex designs, identification of the appropriate level for tests and full reporting of outcomes
☐	☒	Estimates of effect sizes (e.g. Cohen's *d*, Pearson's *r*), indicating how they were calculated

Our web collection on statistics for biologists contains articles on many of the points above.

Software and code

Policy information about availability of computer code

Data collection	No software was used.
Data analysis	ABCD and Generation R: dcm2niix was used for nifti conversion (https://github.com/rordenlab/dcm2niix), fMRIPrep was used for image analysis (https://github.com/nipreps/fmriprep), Python (version 3.9.0) was used to extract connectivity matrices, and R (version 4.1.0) was used for data analysis. All analysis code are publicly available in: https://github.com/EstellaHsu/Brain_dimensions_ABCD_GenR<br><br>Parts of the code were modified based on the code from: (1) https://github.com/dinga92/niclin2019-biotypes, (2) https://github.com/cedricx/sCCA/tree/master/sCCA/code/final

For manuscripts utilizing custom algorithms or software that are central to the research but not yet described in published literature, software must be made available to editors and reviewers. We strongly encourage code deposition in a community repository (e.g. GitHub). See the Nature Portfolio guidelines for submitting code & software for further information.

Data

Policy information about availability of data

All manuscripts must include a data availability statement. This statement should provide the following information, where applicable:

- Accession codes, unique identifiers, or web links for publicly available datasets
- A description of any restrictions on data availability
- For clinical datasets or third party data, please ensure that the statement adheres to our policy

The ABCD data reported in this paper are openly available upon approval from the NDA Data Access Committee. The ABCD data came from ABCD collection 3165 (ABCD BIDS Community Collection (ABCC), https://collection3165.readthedocs.io) and the Annual Release 4.0 (https://doi.org/ 10.15154/1523041).

The Generation R datasets generated and/or analyzed during the current study may be made available upon request to the Director of the Generation R Study, Vincent Jaddoe (v.jaddoe@erasmusmc.nl), in accordance with the local, national, and European Union regulations.

Human research participants

Policy information about studies involving human research participants and Sex and Gender in Research.

Reporting on sex and gender	For all analyses, biological sex (obtained from caregiver-report questionnaires) was used as a covariate. No stratified analyses were run.
Population characteristics	The present study was embedded in two large population-based cohorts, the ABCD Study and the Generation R Study. ABCD: the ABCD Study is a population-based cohort starting from preadolescence until adulthood, which is conducted in 21 study sites across the US. The sample included approximately reflects the sociodemographic variation of the US population. We included the baseline measures (age 9 -11) of brain imaging and behaviors of children. Around 57% of included children were white, 12% African American, 19% Hispanic, 2% Asian, and 10% others. Child biological sex was generally balanced in the current sample (49% girls).<br><br>Generation R: the Generation Study is a prospective population-based cohort from fetal life until adulthood in Rotterdam, the Netherlands. The sample reflects the ethical diversity of Rotterdam. Around 66% included children were Dutch, 17% non-Dutch European, and 17% non-Europeans. Child biological sex was generally balanced in the sample included (52% girls).
Recruitment	ABCD: The primary recruitment approach of the ABCD study is through public and private elementary schools across nationally distributed 21 study sites.<br><br>Generation R: All pregnant women lived within in Rotterdam (defined by postal codes) with a delivery data between April 2002 and January 2006 were invited to participate. Midwives and obstetricians informed eligible mothers about the study at their first prenatal visit in routine care.
Ethics oversight	ABCD: ethical approval was received from the institutional review boards of the University of California (San Diego) and each ABCD site, adhering to their Institutional Review Board approved protocols, state regulations, and local resources.<br><br>Generation R: ethical approval was obtained through the Medical Ethics Committee of Erasmus MC, University Medical Centre (Rotterdam). Informed consent or assent has been received from the included participants.

Note that full information on the approval of the study protocol must also be provided in the manuscript.

Field-specific reporting

Please select the one below that is the best fit for your research. If you are not sure, read the appropriate sections before making your selection.

☐ Life sciences ☒ Behavioural & social sciences ☐ Ecological, evolutionary & environmental sciences

For a reference copy of the document with all sections, see nature.com/documents/nr-reporting-summary-flat.pdf

Behavioural & social sciences study design

All studies must disclose on these points even when the disclosure is negative.

Study description	Cross-sectional study with quantitative data
Research sample	Children sampled from the general population. See above.
Sampling strategy	ABCD: probability sampling of U.S. public and private elementary schools within the 21 catchment areas was used.

Sampling strategy	Generation R: All pregnant women lived within in Rotterdam with a delivery data between April 2002 and January 2006 were invited to participate. All children who visited the MRI at age 9 were eligible for this particular study.
Data collection	ABCD: MRI acquisition was done on children using 3T MRI systems across 21 sites in the US. The behavioral measures (CBCL) and demographic information were assessed by parent-report questionnaires. Child cognitive ability was evaluated by NIH Toolbox of neurobehavioral assessments.<br>Generation R: MRI acquisition was done on children with a single 3T MRI system. The behavioral measurements and demographic information were filled in via maternal-report questionnaires.
Timing	ABCD: the baseline data of ABCD was used in this study. The baseline data collection is from 2017 to 2018.<br>Generation R: the second wave neuroimaging data collected starting from April 2013 to was included.
Data exclusions	ABCD: Of the 9,441 children whose rs-fMRI data were available, we excluded 3,720 children who failed the quality control of the resting-state connectivity data, 220 children with incidental findings, and 14 children with any missingness in behavioral measures and covariates. For families with multiple participants, one twin or sibling was randomly included (595 excluded). Accordingly, data from 4,892 participants, of which around 7.6% had clinically relevant total problem symptom scores, were available for analysis in ABCD.<br><br>Generation R: Among the 3,992 children who were scanned with MRI, 3,289 rs-fMRI scanning were available. We excluded children as a result of the image quality assurance protocol (n=780), and children with higher than 25% missing values in the behavioral assessments (n=358). After randomly including one twin or sibling (n=108), 2,043 participants (around 5.1% were clinically relevant) were included in the final sample for analysis.
Non-participation	ABCD: 11,880 children around age 9 -11 were recruited, and round 9,441 of them have available resting-state functional connectivity data.<br>Generation R: 3,992 children around 9-12 years old were scanned in the second wave neuroimaging data collection. 3,289 of them have the resting-state fMRI scans.
Randomization	Participants were randomly assigned to training and test sets in ABCD based on the study sites.

Reporting for specific materials, systems and methods

We require information from authors about some types of materials, experimental systems and methods used in many studies. Here, indicate whether each material, system or method listed is relevant to your study. If you are not sure if a list item applies to your research, read the appropriate section before selecting a response.

Materials & experimental systems

n/a	Involved in the study
☒	☐ Antibodies
☒	☐ Eukaryotic cell lines
☒	☐ Palaeontology and archaeology
☒	☐ Animals and other organisms
☒	☐ Clinical data
☒	☐ Dual use research of concern

Methods

n/a	Involved in the study
☒	☐ ChIP-seq
☒	☐ Flow cytometry
☐	☒ MRI-based neuroimaging

Magnetic resonance imaging

Experimental design

Design type	resting-state
Design specifications	ABCD: the first 5 min run was acquired for resting-state scans.<br>Generation R: the duration of resting-state scans was 5 minutes 52 seconds for each child.
Behavioral performance measures	This is not applicable for resting-state functional connectivity data.

Acquisition

Imaging type(s)	functional
Field strength	3 Tesla
Sequence & imaging parameters	ABCD: For resting-state data: Siemens (TR = 800 ms, TE = 30 ms, flip angle = 52∘, matrix = 90 × 90, field of view (FOV) = 216 × 216 mm, slice = 60); Philips (TR = 800 ms, TE = 30 ms, flip angle = 52∘, matrix = 90 × 90, field of view (FOV) = 216 × 216 mm, slice = 60); GE (TR = 800 ms, TE = 30 ms, flip angle = 52∘, matrix = 90 × 90, field of view (FOV) = 216 × 216 mm, slice = 60).

Generation R: GE 750, rs-fMRI data were obtained using an interleaved axial echo planar imaging sequence (TR = 1,760 ms, TE = 30 ms, flip angle = 85∘, matrix = 64 × 64, field of view (FOV) = 230 × 230 mm, slice thickness = 4 mm).

Area of acquisition: whole brain

Diffusion MRI: ☐ Used ☒ Not used

Preprocessing

Preprocessing software: FMRIPrep pipeline (ABCD, version 20.2.0, and Generation R, version 20.2.7)

Normalization: Nonlinear registration using the Advanced Normalization Tools (ANTs) was used in both ABCD and Generation R.

Normalization template: MNI space (6th Generation MNI ICBM 152 supplied with FSL)

Noise and artifact removal: Data were adjusted for CSF and white matter signals (plus their temporal derivatives and quadratic terms), low frequency temporal regressors for high-pass temporal filtering, and 24 motion regressors (6 base motion parameters + 6 temporal derivatives + 12 quadratic terms).

Volume censoring: Volumes were not censored, but flagged volumes were added as a regressor to the confounder regressors matrix.

Statistical modeling & inference

Model type and settings: We applied sparse CCA (SCCA), a multivariate unsupervised machine learning technique that can simultaneously evaluate the relationships between two sets of variables from different modalities. SCCA imposes both l1-norm and l2-norm penalty terms, an elastic net regularization combining the LASSO and ridge penalties, to high-dimensional data sets and achieves sparsity of the solution. it aims to find canonical variates (brain-behavior dimensions) that are the low dimensional representation of brain and behavioral covariance.

Effect(s) tested: The canonical correlations between multiple behavioral measures and different resting-state connectivity networks.

Specify type of analysis: ☐ Whole brain ☐ ROI-based ☒ Both

Anatomical location(s): We used regions of interest for the whole cortex and subcortex based on the Gordon parcellation (cortex) and FreeSurfer subcortical parcellation.

Statistic type for inference (See Eklund et al. 2016): We implemented permutation test for the significance tests of canonical correlations obtained from the SCCA model.

Correction: False Discovery Rate (FDR) was used to correct for multiple testing of canonical correlations

Models & analysis

n/a	Involved in the study
☐	☒ Functional and/or effective connectivity
☒	☐ Graph analysis
☐	☒ Multivariate modeling or predictive analysis

Functional and/or effective connectivity: fisher r-to-z transformed connectivity matrices calculated by Pearson correlation.

Multivariate modeling and predictive analysis: Input variables: The input of the model is the CBCL scores (psychiatric symptoms) and brain connectivity features (the first 100 principal components).

Dimensionality reduction: prior to SCCA analysis, the upper triangle of the 349 × 349 functional connectivity matrix was flattened and residualized by covariates. Principal Component Analysis (PCA) with a weighting scheme was applied to reduce the connectivity features into principal components (PCs) that aggregated the information of the data.

Training and test sets: we used ABCD as the discovery set (n=4,892), in which all analyses were conducted (trained) and tested. The ABCD discovery set was randomly split into a training set consisting of 18 sites and a test set consisting of 3 sites. To reduce sampling biases, the split procedure was repeated 30 times, resulting in 30 pairs of independent train-test sets. Importantly, the analyses in ABCD Training sets and ABCD Test sets were fully separated to safeguard the results from data leakage. In a final step, Generation R was used as an independent external validation set (N=2,043). We characterized two approaches of out-of-study generalizability tests: (1) qualitative replication (simply repeating the analysis and train a new model), and (2) the gold-standard test (applying the model weights from ABCD to Generation R).
